# The Histological Composition of Capsular Contracture Focussed on the Inner Layer of the Capsule: An Intra-Donor Baker-I Versus Baker-IV Comparison

**DOI:** 10.1007/s00266-018-1211-1

**Published:** 2018-09-05

**Authors:** E. de Bakker, L. J. van den Broek, M. J. P. F. Ritt, S. Gibbs, F. B. Niessen

**Affiliations:** 1 Department of Plastic, Reconstructive and Hand Surgery, Amsterdam UMC, Vrije Universiteit Amsterdam, Amsterdam Movement Sciences, P.O. Box 7057, 1007 MB Amsterdam, The Netherlands; 2 Department of Dermatology, Amsterdam UMC, Vrije Universiteit Amsterdam, Amsterdam Movement Sciences, Amsterdam, The Netherlands; 3 Department of Oral Cell Biology, Academic Centre for Dentistry Amsterdam (ACTA), University of Amsterdam and Vrije Universiteit Amsterdam, Amsterdam Movement Sciences, Amsterdam, The Netherlands

**Keywords:** Capsular contracture, Immunohistology, Histology, Synovial metaplasia, Breast reconstruction, Breast augmentation

## Abstract

**Background:**

Capsular contracture remains one of the major complications after breast implantation surgery. The extent of capsular contraction is scored using the Baker scale. The aim of this study was to compare intra-individual Baker-I with Baker-IV capsules, and in particular the prevalence and histological properties of the inner capsule layer.

**Methods:**

Twenty capsules from ten patients were included after bilateral explantation surgery due to unilateral capsular contracture (Baker-IV) after cosmetic augmentation with textured implants. All capsules underwent (immune-)histochemical analysis: haematoxylin–eosin (morphology), CD68 (macrophages), cytokeratin (epithelial cells) and vimentin (fibroblasts), and were visually scored for cell density and the presence of an inner layer and measured for thickness.

**Results:**

Baker-IV (*n* = 10) capsules were significantly thicker compared to Baker-I (*n *= 10) capsules (*P* = 0.004). An inner layer was present in 8 Baker-I capsules. All Baker-I capsules were vimentin and CD68-positive and cytokeratin-negative. Positive vimentin was seen throughout the inner layer, and CD-68 staining was observed adjacent to the intermediate capsule layer. In contrast, only 2 Baker-IV capsules had an inner layer, of which only 1 showed the same profile as Baker-I capsules (*P* = 0.016). No cytokeratin positivity was seen in any capsule. In Baker-IV capsules, outer layers showed more positivity for both vimentin and CD68.

**Conclusion:**

The inner layer is morphologically consistent with synovial metaplasia and is more prevalent in healthy, uncontracted Baker-I capsules. This inverse relation between the presence of the inner layer and higher Baker classification or pathological contracture could indicate a protective role of the inner layer against capsular contracture formation.

**Level of Evidence III:**

This journal requires that authors assign a level of evidence to each article. For a full description of these Evidence-Based Medicine ratings, please refer to the Table of Contents or the online Instructions to Authors www.springer.com/00266.

## Introduction

Since the first silicone breast implants were used in surgery, capsular contracture has been a serious adverse outcome resulting in significant aesthetic and functional complications [1]. Capsular contraction is therefore the subject of numerous studies to understand and prevent its formation [2–10]. The type of implant, implantation technique and post-operative radiotherapy are all factors described to influence the formation of capsular contracture [11]. One of the most striking aspects of capsular contracture is that some patients develop unilateral contracture even though both breasts have been augmented or reconstructed in a similar fashion during the same procedure [12–14]. The capsules of these patients could provide useful intra-donor comparison opportunities, but until now these have not been explored. The pathohistological mechanisms involved in capsular contracture remain unclear despite numerous studies [1, 12, 15–19]. Histologically, the capsules have been described as consisting of 3 layers: an inner layer adjacent to the implant consisting of fibrocytes and histiocytes which forms an epithelial-like or pseudo-epithelial layer (PSE), an intermediate layer of smaller fibrils in a vessel-rich network and an outer collagen- dense layer [17]. Furthermore, this PSE is not consistently reported although there appears to be a higher prevalence with textured implants [20, 21]. While not used as frequently as textured or smooth implants, polyurethane foam-coated implants have been reported to generate a similar layer [22]. More recently, the inner layer has been identified as a metaplasia like synovial layer [3, 9, 24] consisting of macrophages and fibroblasts [23], and at this point, both names are used to describe the same layer [21]. Since very few studies report immunohistochemical stainings targeted specifically at the inner layer, histological information on this specific layer is limited [18, 20, 21, 24–26]. Therefore, the aim of this study was to compare, for the first time, intra-individual Baker-I with Baker-IV capsules. In particular, the focus will be on the prevalence and (immuno-)histological properties of the inner capsule layer and to determine whether this is indeed a metaplasia-like synovial layer or has more epithelial-like characteristics.

## Materials and Methods

### Patients and Tissue Collection

Donor-matched Baker-I and Baker-IV capsules were collected from patients undergoing explantation surgery between 2010 and 2014 due to capsular contracture complaints. All capsules were obtained from adult women who primarily underwent plastic surgery for cosmetic breast augmentation and subsequently developed unilateral complaints, i.e. Baker-I on the one side and Baker-IV on the other. Only cosmetic breast augmentation patients were included to exclude any effects that the breast cancer treatment can have on the tissue. All patients had received high cohesive gel-textured implants in the past with the submuscular method in various clinics within the Netherlands. All complaints were graded with the Baker classification scale, and only matched capsules of patients with Baker-I and Baker-IV grade were included. The discarded capsule tissue was coded to enable the collection of additional relevant information (e.g. type and size of implant, duration of implant placement, age of patient and comorbidity). Clinical grading, the explantation of implants and the collection of capsules, was performed by an experienced plastic surgeon (FBN) and included only after oral informed consent. Tissue collection procedures were performed in compliance with the ‘Code for Proper Secondary Use of Human tissue’ as formulated by the Dutch Federation of Medical Scientific Organization. In total, 10 patients and 20 capsules were included. Patients who had received PIP implants or with a history of (breast) cancer were excluded. Patient characteristics at inclusion are shown in Table 1.

**Table 1:** Patient characteristics

Number of patients	10
Age (years)	47.5 ± 8.9
BMI	24.2 ± 4.1
Duration of implant placement (months)	161 ± 71
Size of implant (cc)	306 ± 10.5
Smoker	3/10
Diabetic	2/10
Tear of implant on Baker-IV side	3/10
Tear of implant on Baker-I side	1/10
More than one augmentation	1/10

Mean ± SD is shown. Tear in implant seen during explantation

### Histological Analysis

Tissue samples were fixed in 4% formaldehyde for 24 h, then routinely processed and embedded in paraffin. Sections of 5 µm were then used for haematoxylin and eosin (HE) staining [27]. The HE-stained capsules were photographed at tenfold magnification (Nikon Eclipse 80i, Düsseldorf, Germany). The thickness was quantified using NIS-Elements AR 2.10 software (Nikon). Of each capsule, at least 5 measurements were made to obtain a representative mean thickness of the capsule. A visual separation was made between the inner, intermediate and outer layers. Each capsule was scored visually for the presence of a visible inner, pseudo-epithelial layer or synovium-like, layer. Cell density was scored for the two outer layers because of the denser nature of an inner layer. Scoring was done independently by two authors.

### Immunohistochemical Analysis

Immunohistochemical analysis was performed to ascertain the composition of the inner layer and to determine whether it had a more epithelial-like or a more synovial-like phenotype where there are type A cells (macrophages) and type B cells (fibroblast-like) present while epithelial-like cells should be cytokeratin positive. Immunohistochemical staining on paraffin-embedded 5-µm sections was performed with cytokeratin, vimentin and CD68 as described previously [28] (all Dako/Agilent, Santa Clara, California, USA, dilution 1:100). For each staining, a positive control normal human skin sample was included. Quantification for positivity was done visually and independently by two authors, noting whether the positivity was in the inner layer or a different part of the capsule and whether the sample showed no, a little, intermediate or a lot of positive cells.

### Statistical Analysis

All mean thickness results were paired for each patient and were analysed with the Student’s two-tailed *t* test for paired variables. The related-samples McNemar test was used to determine correlation between Baker score and the presence of an inner layer and presence of an inner layer with a synovial metaplasia-like phenotype. A *p* value of less than 0.05 was considered significant. All statistics were performed with IBM SPSS Statistics for Windows version 22.0 (IBM Corp. Released 2013. Armonk, NY: IBM Corp.)

## Results

### Patient Characteristics

In total, 10 patients with a Baker-I and Baker-IV capsule were included. In total, 20 capsules were analysed. The mean duration of implantation was 156 months; 3 were smokers and 2 had type II diabetes (Table 1). Out of all explanted implants, 4 had signs of a tear, out of which 3 were on the affected, Baker-IV, side. One patient had a breast augmentation prior to the one of which the capsules were removed for this study. The prior augmentation was also revised because of capsular contracture.

### Capsular Thickness and Morphology

**Fig. 1:**
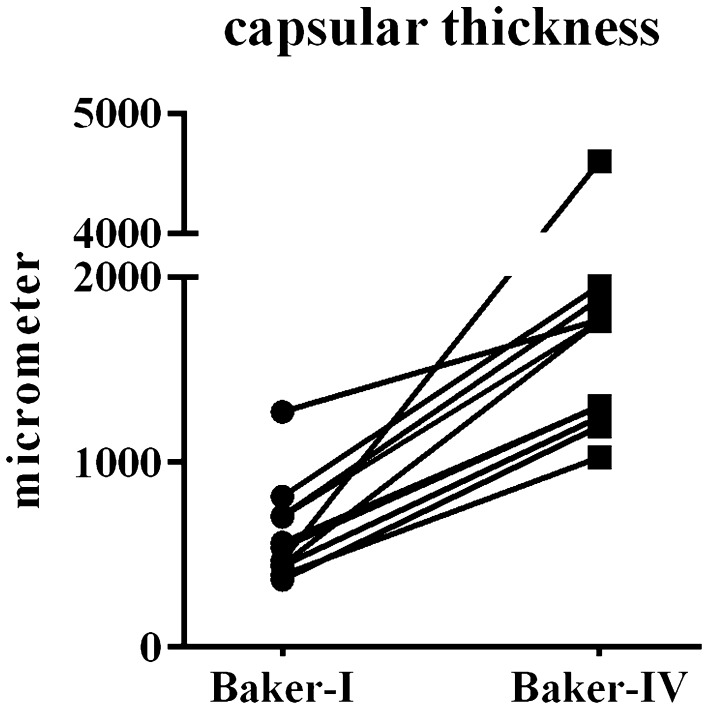
Intra-donor differences in Baker-I and Baker-IV capsular thickness. Average of 5 different measurements within a tissue section is shown for each capsule. Each line represents one donor with a Baker-I and a Baker-IV capsule. Thickness is shown in micrometre (µm)

**Fig. 2:**
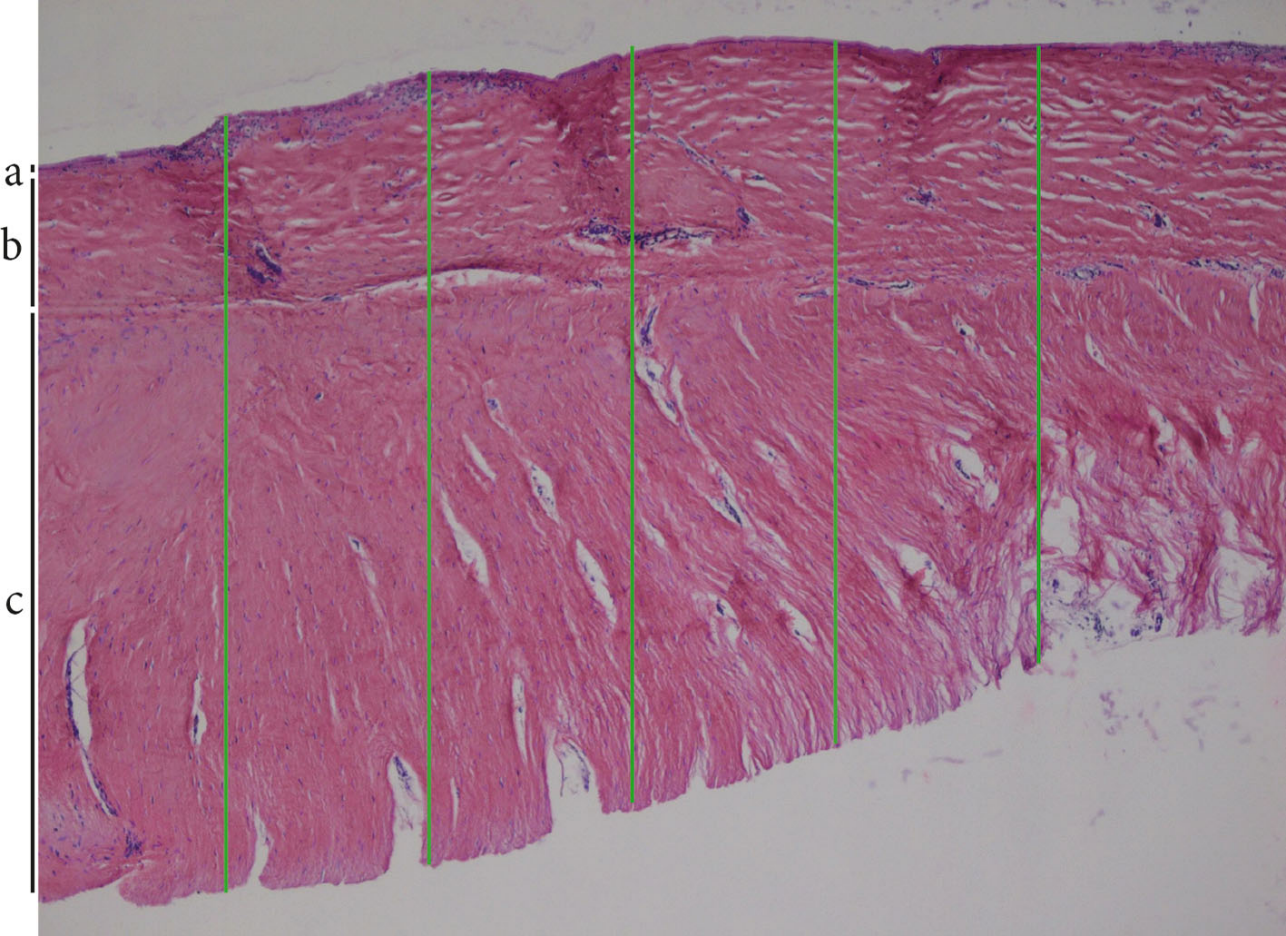
Capsule thickness measurement and layers. Representative photograph of Baker-IV capsule is shown with **a** inner layer, **b** intermediate layer—note extracellular matrix is aligned in line with the implant and **c** outer layer—note extracellular matrix is aligned perpendicular with the implant. The 5 thickness measurements used in Fig. 1 are indicated with green bars

**Fig. 3:**
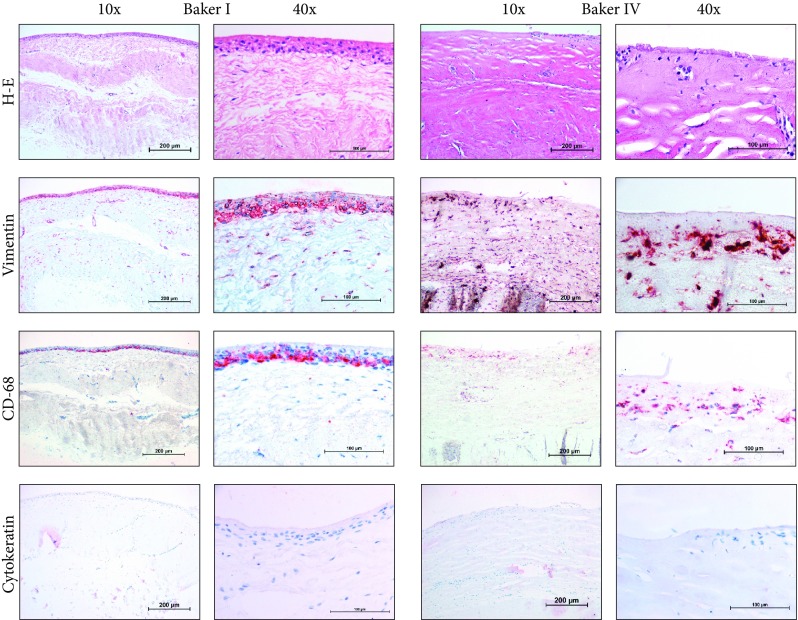
(Immuno-)histochemical staining results of a representative Baker-I and Baker-IV capsule from the same donor. Shown are × 10 and × 40 magnifications

In general, a large variation between samples was observed, both between patients and Baker classification. This included variance between cell density, capsule thickness and organisation. The thickness of capsules was determined by the assessment of HE-stained tissue sections (Fig. 1). The Baker-IV capsules were on average 3.3-fold thicker in comparison with the Baker-I capsules (*P* = 0.004, Table 2). The increase in thickness was comparable for all paired capsules as visually shown in Fig. 1. All capsules were organised in multiple layers (Fig. 2). Some of the capsules containing an internal layer, directly adjacent to the implant, consisting of cells was arranged in a palisaded manner (Figs. 2, 3). In the current literature, this inner layer is sometimes called a pseudo-epithelial or synovial metaplasia-like layer. Beyond this layer, more outer wards, all capsules contained two thicker layers. The intermediate layer was generally organised in line with the border of the capsule. The outermost layer appears more loosely organised and is aligned perpendicular to the implant (Fig. 2). The Baker-I capsules more frequently showed the inner layer (8/10) compared to Baker-IV capsules (2/10) (Fig. 3 and Table 2). The McNemar test showed that this result was significant (*P* = 0.03, Table 2). When an inner layer was present in the Baker-IV capsule, it was less consistent in nature and not present on the entire border of the capsule. In contrast, most Baker-I capsules showed very consistent and continuous inner layers (Fig. 3). As can clearly be seen in Fig. 3, an inner layer is an extremely cell-dense region consisting of up to four stratified cell layers. In contrast, the outer layers consisted of single cells dispersed throughout the tissue. In cell density, there was again a large variance between Baker-I and Baker-IV capsules. In general, cell density is higher for the Baker-IV capsules (Table 2).

**Table 2:** Capsule characteristics

Characteristic	B1	B4	*P*
Entire capsule			
Thickness	543 ± 152 µm	1802 ± 1035 µm	0.004
Inner layer			
Present	Y (8/10)	Y (2/10)	0.031
Vimentin	Y (8/8)	Y (1/2)	
CD68	Y (8/8)	Y (2/2)	
Cytokeratin	N (8/8)	N (2/2)	
Vim +/CD68 +/Cytokeratin-	8/10	1/10	0.016
Intermediate and outer layer			
Vimentin	+(3/10)	+(0/10)	
	++(3/10)	++(4/10)	
	+++(4/10)	+++(6/10)	
CD68	+(8/10)	+(5/10)	
	++(1/10)	++(3/10)	
	+++(1/10)	+++(2/10)	
Cytokeratin	−(10/10)	−(10/10)	
Cell density	+(7/10)	+(3/10)	
	++(2/10)	++(6/10)	
	+++(1/10)	+++(1/10)	

Summary of morphological and histological characteristics of the capsules. Thickness in µm ± standard deviation, measured at fourfold magnification using NIS-Elements AR 2.10 software (Nikon). In each photo, at least 5 measurements were made. Cell density, inner layer presence and positive staining scored visually by two independent authors noting if an inner layer was present (Y), if that layer showed positivity (Y) or not (N) and for the outer layer, if it had low (+), medium (++) or high cell density (+++), positivity for the staining and whether it showed no (−), low (+), intermediate (++) or a lot (+++) of positive cells

### Phenotype of Cells

Next, the phenotype of the inner layer was determined. Cytokeratin, which identifies epithelial cells, was negative in all capsules while being positive in our control skin sample. This implied that a synovial metaplasia may be present [29]. Out of the 10 Baker-I capsules, 8 showed an inner layer out of which all were indeed vimentin and CD68-positive and cytokeratin-negative. These inner layers showed positive vimentin throughout the inner layer, and CD-68 staining was observed in the lowest cell layer adjacent to the intermediate capsule layer (Table 2; Fig. 3). In contrast, in the Baker-IV capsules, only 2 capsules had an inner layer out of which only 1 showed the same profile as the Baker-I capsules; this is a statistically significant result (*P* = 0.016, Table 2). Finally, the phenotype of the outer layers was determined. In nearly all capsules, vimentin positive cells were observed as single cells throughout the intermediate and outer capsule layers (Table 2, Fig. 3). Overall, there were more vimentin positive cells in the Baker-IV capsules compared to Baker-I. With regard to CD68, only very few cells stained positive in the outer layer of Baker-I, in contrast to the densely stained inner cell layer. For Baker-IV, positive-staining cells were found throughout the intermediate and outer layers. No significant correlation was found between the presence of an inner layer and positivity for CD68 or vimentin in the outer layers.

## Conclusion

These findings suggest that a synovial metaplasia-like inner layer is more often present than previously thought and is more prevalent in healthy, uncontracted Baker-I capsules. Morphologically, this layer is consistent with a synovial metaplasia-like nature rather than an epithelial-like layer. There is an inverse relation between the presence of this inner layer and higher Baker classification or pathological contracture, and it could therefore play a protective role against the formation of capsular contracture. A clear relation between Baker grade and the presence of a synovial metaplasia-like layer has not been found until now. Future research should focus on the mechanism of loss of this inner layer and whether preventing the loss of this layer could prevent capsular contracture.

## Discussion

This present study highlights the differences between Baker-I and Baker-IV capsules within the same donor. All patients had developed unilateral local contracture complaints, which is one of the most striking characteristics of the aetiology of capsular contracture. For the first time, these differences are shown in a donor-matched group. We also show that even within the same donor, there can be a large variance in morphology of the capsules. The finding of a significant difference in capsule thickness reaffirms earlier studies [30]. This study shows a significant difference between the Baker-I and Baker-IV capsules concerning the presence and form of a synovial metaplasia-like layer. This inner layer, when present, consists of vimentin (mesenchymal cells) and CD68 (macrophages)-positive and cytokeratin (epithelial)-negative cells, closely stacked together towards the implant in a palisaded manner. This cytology is in line with earlier publications about synovial metaplasia in breast implant capsules and is also found with other foreign body reactions [31]. Because the synovial metaplasia-like layer in the Baker-IV capsules is less congruent in comparison with the Baker-I capsules, it could be hypothesised that these are remnants of previously intact synovial metaplasia-like layers. It could be possible that this inner layer either fulfils a protective role against contracture and that, when lost, the continuous contact with the prosthesis activates a secondary pathway leading to contracture. It is also possible that this layer is lost due to the increasing amount of tissue in the outer layers and the loss of nutrition to the inner layer. A theory is that this inner layer is a specialised form of repair and disappears over time [29]. However, since the average duration of implant placement in this study is 13 years with the inner layer still showing in the Baker-I capsules, it seems unlikely that this layer would disappear over time. The stronger overall positivity in all layers for vimentin could correlate with more fibroblasts present in the Baker-IV capsules. Fibroblasts are responsible for production of extracellular matrix, and this study showed that Baker-IV capsules are significantly thicker than Baker-I capsules.

Macrophages play a key role to stimulate fibroblasts in the formation of granulation tissue and fibrosis, and their role in foreign body reactions is a very actual topic [32]. While macrophage presence and key role seem evident, different subtypes and reactions might be causal to the actual formation of capsular contracture. If the macrophage in the Baker-I capsule is anti-fibrotic and protective (M1), in the Baker-IV capsule, this role could be reversed (M2). In our experience, there are cases in which women had experienced an event which might have led to this shift in behaviour of the cells surrounding the implant such as direct trauma to the breast or pregnancy. This repeated exposure of the implant to stress could be the start of a more, secondary, profibrotic process. The macrophage seems to be susceptible to modulation and has already been suggested as a target for a therapeutic approach to prevent capsular contraction [33]. Our results indicate only a slight increase in CD68 positivity in the outer layers of the Baker-IV capsules, and therefore, it is likely that one or more other types of immune cells, like different T-cell subsets, are also involved in this process [34]. This study only included capsules of textured implants due to the fact that these are used nearly exclusively in the Netherlands and a comparison with capsules of smooth implants should be pursued in the future.
